# Cubosomes in Drug Delivery—A Comprehensive Review on Its Structural Components, Preparation Techniques and Therapeutic Applications

**DOI:** 10.3390/biomedicines11041114

**Published:** 2023-04-07

**Authors:** Durgaramani Sivadasan, Muhammad H. Sultan, Saad S. Alqahtani, Shamama Javed

**Affiliations:** 1 Department of Pharmaceutics, College of Pharmacy, Jazan University, Jazan 45142, Saudi Arabia; mhsultan@jazanu.edu.sa (M.H.S.); sjahmad@jazanu.edu.sa (S.J.); 2 Department of Clinical Pharmacy, College of Pharmacy, Jazan University, Jazan 45142, Saudi Arabia; ssalqahtani@jazanu.edu.sa

**Keywords:** cubosomes, characterisation, nanomedicine, drug delivery systems

## Abstract

Cubosomes are lipid vesicles that are comparable to vesicular systems like liposomes. Cubosomes are created with certain amphiphilic lipids in the presence of a suitable stabiliser. Since its discovery and designation, self-assembled cubosomes as active drug delivery vehicles have drawn much attention and interest. Oral, ocular, transdermal, and chemotherapeutic are just a few of the drug delivery methods in which they are used. Cubosomes show tremendous potential in drug nanoformulations for cancer therapeutics because of their prospective advantages, which include high drug dispersal due to the structure of the cubic, large surface area, a relatively simple manufacturing process, biodegradability, ability to encapsulate hydrophobic, hydrophilic, and amphiphilic compounds, targeted and controlled release of bioactive agents, and biodegradability of lipids. The most typical technique of preparation is the simple emulsification of a monoglyceride with a polymer, followed by sonication and homogenisation. Top-down and bottom-up are two different sorts of preparation techniques. This review will critically analyse the composition, preparation techniques, drug encapsulation approaches, drug loading, release mechanism and applications relevant to cubosomes. Furthermore, the challenges faced in optimising various parameters to enhance the loading capacities and future potentialities are also addressed.

## 1. Introduction

Drug delivery systems are devices that transport a therapeutic agent to a specific site inside the body. Controlled drug release is pre-designed to achieve effective concentration at the site of action, thus minimising toxic side effects while promoting therapeutic benefits. A more evolved system of the same, termed novel drug delivery system (NDDS), is the growing trend in the current drug delivery scenario, mostly as a result of the fact that they address the limitations of traditional drug delivery systems. Their tangible benefits include reduced dosing frequency, enhanced site-specificity, reduced toxic side effects, degradation resistance specifically against the acidic gastric environment and increased bioavailability. Findings suggest that NDDS are a promising option to tackle major diseases. In light of this, several different types of carriers have been developed in the past decade, and newer ones are being developed at a phenomenal pace [1,2].

Drug delivery improves a drug’s efficacy. However, to curate optimal drug release profiles, the rate of active release must be controlled, as well as the ease of preparation and vehicle stability. All of these attributes must be successfully incorporated into an ideal delivery vehicle. Surfactants and polymers are extensively used in the production of guided drug delivery systems. They generate supra-assemblies, which are widely used as effective delivery modules. Cross-linked gel networks or liquid-crystalline aggregates are examples of these systems, which load, stabilise, and finally distribute active components [3]. Nanomedicine and nano-delivery systems are rapidly growing disciplines in which microscopic materials are employed as diagnostic tools or to deliver therapeutic drugs to specific sites in a controlled manner. Nanotechnology can help treat chronic human diseases by allowing precise medicines to be delivered to specific locations, a process known as target-oriented delivery. There have been several noteworthy applications of nanomedicine, such as chemotherapeutic agents, immunotherapeutic agents, biological agents, etc., in the treatment of various diseases in recent years [4]. Small nanospheres made out of materials chosen at the atomic or molecular level are known as nanoparticles. Thus, they can pass through the human body more easily than larger materials. Nanoscale particles have distinct biological and structural features. Nanomedicines have attracted attention in recent years as a result of their potential to encapsulate pharmaceuticals or bind therapeutic chemicals to nanostructures and deliver them to specific tissues with greater precision and control. It refers to the use of nanoscale materials in live cells, such as nano-sensors, nanorobots, and actuation materials for diagnosis, conveyance, and sensory functions [5,6].

The therapeutic actions of all forms of delivery systems for drugs, including liposomes, are influenced by drug release rates. Liposomes are tiny phospholipid vesicles that can be exploited to circumvent several medications’ putative barriers for successful delivery to their target tissues, like tumours [7]. Liposomes were the first closed bilayer phospholipid structures to be developed in 1965, and they were quickly proposed as medication delivery methods. Liposomes were the first pioneer drug delivery systems to reveal that they could alter the in vivo dispersion of encapsulated medicines. Simultaneously, novel technologies for preparing large unilamellar liposomes (LUV) with improved trapping effectiveness and uniformity were also developed. Liposomes were created in a low-pressure, low-throughput manner as originally envisioned, and higher-pressure systems were developed afterwards to accomplish larger-scale manufacturing [7]. Drug retention capabilities in liposomes vary depending on the drug; some medicines, such as doxorubicin, precipitate rapidly inside liposomes after assemblage and have exceptional retention properties, while others, for example, ciprofloxacin, do not precipitate readily and are more challenging to retain [8,9]. Drug retention can be boosted by loading pharmaceuticals to obtain optimum intraliposomal drug concentrations exceeding their solubility limits, resulting in increased precipitation, or by encapsulating polyanions, including dextran sulphate [10].

The use of ‘classical’ and ‘stealth’ liposomes as sustained-release drug delivery devices for in vivo delivery of therapeutics, from monomeric medicines to nucleic acids, has become ubiquitous. In an earlier investigation involving a small number of patients, long-circulating (PEGylated) liposomes were found to accumulate extensively in Kaposi’s sarcoma and malignancies of the head and neck, with intermediate in lung cancer and lesser in breast cancer [7,11]. Although several modes of delivery have been employed for liposomal and lipid-based medicines, parenteral administration, particularly intravenous administration, is the most common for clinically approved products. Because the gastrointestinal breakdown of the carrier results in poor bioavailability of linked medicines, oral administration is not commonly used for liposomal products [11]. Marqibo^®^, a liposomal preparation of vincristine licensed in August 2012 to treat acute lymphoblastic leukaemia at second or higher recurrence, is the latest liposomal medication to earn Food and Drug Administration (FDA) clearance [12]. The difference between liposomes and cubosomes is shown in Table 1.

Cubosomes are biocompatible carriers in drug delivery that are nanostructured liquid-crystalline particles formed of specific amphiphilic lipids in different ratios. Cubosomes are bicontinuous cubic phases that have already been reversed and have unique physicochemical characteristics [13]. These unique systems are a study area of interest because they can provide a vast range of hydrophobic, hydrophilic, and amphiphilic medicines, having improved bioavailability and loading potential. They are employed frequently in different drug delivery applications, including oral, transdermal, ocular, and chemotherapy. Because of their enormous application as an alternate drug delivery mechanism to liposomes, cubosomes have been regarded as the drug nanocarrier in recent years. Monoolein–water cubosomes, especially those comprised of binary systems, can self-assemble into cubic crystalline forms, capable of being thermodynamically stable [14]. The relevant literature on cubosomes will be critically evaluated in this review, with emphasis on the advantages of cubosomes, along with their structure and composition, methods of preparation and drug delivery applications.

**Table 1:** Difference between cubosomes and liposomes [15].

Cubosomes	Liposomes
Distinct, submicron, nanostructured particles of bicontinuous cubic liquid-crystalline phase enclosing two separate regions of water divided by surfactant-controlled bilayers.	Spherical vesicles have an aqueous core enclosed by one or more phospholipid bilayers. The main components are cholesterol and phospholipids.
Retain their stability even at high dilution, which is not possible with other liquid-crystalline systems. Higher ratio of particle volume and bilayer area in comparison with the liposomes.	They contain biocompatible and biodegradable lipids and are inert and non-immunogenic.
Encapsulate all three types of hydrophilic, hydrophobic and amphiphilic substances.	Can be loaded with hydrophilic and hydrophobic molecules.
Better stability than liposomes and, due to their liquid-crystalline membrane architecture, possess a greater ability to envelop and encapsulate hydrophobic chemotherapeutic agents. Extremely high encapsulation efficiency and enhanced apoptotic efficacy.	Sometimes phospholipid undergoes oxidation and hydrolysis-like reaction.
High-energy methods such as ultrasonication, homogenisation and micro-fluidisation are used to prepare cubosomes.	Provide selective passive targeting to tumour tissues. Flexibility to couple with site-specific ligands to achieve active targeting. Prepared by physical dispersion, solvent dispersion and detergent solubilisation technique.
Challenges faced in optimising various parameters to enhance the loading capacities and subsequent improvement in their release are a few limitations of these novel delivery systems.	Chances for leakage and fusion of encapsulated drugs. High production cost, low solubility with a short half-life.

The cytotoxicity of cubosomes depends on several factors, such as internal nanostructures, lipid chemistry, and the type of stabilisers used. A study revealed that the cubosomes prepared with poly(phosphoester) (PPE), a structural analogue of the traditional F127, were significantly less toxic than carriers containing F127. This result was observed and evaluated in the cell lines human embryonic kidney 293 (HEK-293) and Human umbilical vein endothelial cells (HUVEC). It was also demonstrated that the PPE-based formulation has a high hemocompatibility in contrast to cubosomes prepared with F127, which reveals a certain degree of cytotoxicity against erythrocytes. Furthermore, several studies reported a half maximal inhibitory concentration (IC50) value of monoolein-based cubosomes in the range of 30–100 µg/mL. Cubosomes are included in the US FDA’s list of inactive ingredient guidelines [16].

## 2. Cubosomes and Their Types

Amphiphilic Bicontinuous Cubic Phases or BCPs aggregate in selected solvents to form a wide variety of morphologies, including spheres, cylinders, vesicles (polymersomes), ribbons, films, fibres, tubules, multi-geometry nanoparticles driven by the reduction of energetically unfavourable segment/solvent interactions. Bicontinuous mesostructures are a subclass of these morphologies that are distinguished by their 3D percolating phase structure. Although bicontinuous phase structures are well-known, their stability range is limited. Dispersed synthetic particles containing bicontinuous cubic liquid-crystalline nanostructures, also known as cubosomes, are usually used for low molecular weight surfactants [15]. Cubosomes are a versatile novel drug delivery platform with a high loading potential of actives and their sustained release, possessing different physiochemical properties. They are nanostructured entities with cubic crystallographic symmetry, similar in structure to the parent phase but with a substantially higher surface area and lower viscosity. This nanodispersion is a viable method for overcoming the cubic phase’s main disadvantages [13]. Amphiphilic lipids such as glycerol monooleate (GMO) with the capability of self-assembling in liquid conditions are used to create cubosomes. They have a three-dimensional structure comparable to a honeycomb (100–500 nm) [17].

X-ray scattering measurements were used by Luzzati et al. to discover the existence of cubic phases in the lipid-water system [18]. Fontell et al. reached similar conclusions in parallel about the cubic phase in ternary systems of amphiphiles, oils, and water, although not being aware of the lipid work [19]. Lutton published a comprehensive investigation of monoglyceride aqueous phase behaviour around the same period. Monoglycerides are polar lipids with low water solubility that behave in the aqueous phase due to their structural resemblance to non-ionic surfactants [16]. In the 1980s, Kre Larsson wrote a review on cubic lipid/water phases, which followed the findings of Patton and Carey, who had studied the formation of bicontinuous cubic compositions as a product of lipid degradation [20]. The creation of colloidal dispersions of non-lamellar lyotropic crystalline phases has been patented by Landh and Larsson, who have dubbed the particles “cubosomes”. Larsson pioneered work on cubic structures, discovering that cubosomes can be created from bulk cubic structures when they are dispersed in aqueous conditions, forming submicron particles with an identical interior to the parent structure [21].

**Figure 1:**
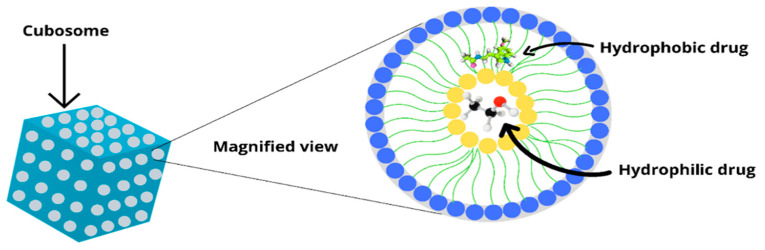
Structure of a cubosome.

Cubosome particles are made by mechanically fragmenting the cubic lipid-water phase in a three-phase region containing a liposomal dispersion, and they are called cubosomes to distinguish them from liposomes. Its structure differs from liposomes in that it may accommodate lipid-soluble, water-soluble, and amphiphilic molecules simultaneously. Cubosomes are thermodynamically stable and can persist for an infinite amount of time. The addition of polymers to cubosome colloidal dispersions can help to stabilise them. They also have the potential for regulated delivery of actives, as the tortuous diffusion of the actives through the cubic phase’s “regular” channel structure governs diffusion. They form in aqueous surfactant systems at high amphiphile concentrations and have enough relative molecular alignment to be distinguished by geometric symmetry [17]. Based on differential geometry principles, open and closed cubosome structures can be defined. The open cubosome has both aqueous channels connecting the exterior environment, whereas the closed cubosome has one water channel open to the exterior and the other in isolation, as seen in Figure 1. Cubosomes are categorised as a gyroid, primitive, or diamond, and they maintain cubic symmetry like their bulk parent phase [15,22,23].

## 3. Theories on Cubic Phase Structure

Cubosomes or bicontinuous cubic phase liquid crystals have several features that are intriguing as a generic medication delivery system. It is formed into bilayers inside the surfactant and wrapped into a three-dimensional, periodic, and minimum surface, generating a densely packed structure. The material is an optically transparent, very viscous bicontinuous cubic liquid-crystalline phase with a unique structure in the nanometer range. They are relatively easy to make, and the improved penetration power and emulsification properties of lipids allow them to encapsulate hydrophobic, hydrophilic, and amphiphilic compounds while ensuring the targeted and controlled release of bioactive compounds [24]. The three macroscopic phases of the cubic structure that are often seen during cubosome synthesis are the precursor, bulk gel, and particle dispersion phases. A solid or semisolid material that produces the cubic phase in reaction to stimuli, including contacting a liquid, is designated the precursor state. The bulk gel-cubic phase, on the other hand, is rigid, isotropic, and can be expanded into cubosomes. Finally, the dispersion of the solid-like phase into smaller particles forms cubosomes [25].

### 3.1. Fontell & Drew Theory

Cubic phases can be found in ternary systems of amphiphiles, oil and water, and various monoglycerides. Monoglycerides are polar lipids with low water solubility and aqueous phase behaviour that is structurally similar to non-ionic surfactants. Lutton’s results show that monoglycerides with hydrocarbon chain lengths between C-12 and C-22, particularly monoolein, have a bigger cubic phase area. Monoolein, also known as C-18 Monoglycerides, is an unsaturated fatty acid [19,26].

### 3.2. Gustafson et al. Theory

Cubosomes are single-crystal formations with unilamellar vesicles visible and distributed lamellar liquid-crystalline phase particles. The formation of larger vesicles is aided by increasing the polymer-to-monoolein ratio [19]. Slow transport processes that form highly viscous crystalline structures and the high energy required for fragmentation result in mostly vesicles through ultrasonication of bulk cubic phases that are trace formed into cubosomes via membrane fusion over time. This metastability is one of the many characteristics of cubosomes systems (bulk cubic phase). Cubosomes are also colloidally stabilised by vesicles [27].

### 3.3. Schwarz, Jacob & Anderson Theory

In non-ionic surfactant systems, cubic phases are frequently encountered wedged between lamellar and hexagonal liquid-crystalline phases. The monoolein-water system is remarkable in that it has a cubic phase area with a wide range of composition and temperature. Surfactant packing concepts, on the other hand, are getting closer. Normally, monoolein has a hydrophilic head and a hydrophobic tail, resulting in reversed or inversed cubic phases, indicating polar medium phases. As a result, cubic phase structures can be represented using differential geometry and periodic minimum surfaces. The ideal way to characterise minimal surfaces is to compare them to soap films. Three types of minimum surfaces are investigated in cubic phases based on their curvatures. At high water levels, the monoolein-water system creates the D-surface, and at lower water levels, the G surface. The p-surface forms in the monoolein-water system, but only when a third component, such as caseins or amphiphilic molecules, is present. The block copolymer is incorporated. The existence of cubic phases can be determined using the X-ray scattering technique. Cubosomes are visualised using transmission electron microscopy (TEM) and freeze-fracture electron microscopy [27].

### 3.4. System Forming Theory

Cubosomes can form in binary and ternary systems if the cubic phase and the solvent have a significant miscibility gap. When poloxamer 407 is employed to prevent cubosome aggregation and flocculation, the cubosomes have good colloidal stability. They can be encased in lamellar bilayer caps, which seal the cubic bilayer opening created by fragmentation and offer colloidal stability by preventing hydrocarbon chains from coming into contact with water. The colloidal stability of cubosomes coated with a solid crystalline bilayer is better, whereas lamellar liquid-crystalline coatings are rigid. In addition, sponge phase coatings as a cubosome stabilising coating have been proposed. Another molecule with a high potential for cubosome development is phytonadione [27].

## 4. Mechanism of Drug Release from Cubosomes

The drug release mechanism from cubosomes is based on the principle of drug diffusion, where the concentration gradient of the drug across the cubosomes is the driving force of the diffusion. Therefore, the drug release rate from cubosomes is generally coincidental with the Higuchi or Fick diffusion equation. There are many factors influencing the drug release rate, such as drug solubility, diffusion coefficient, partition coefficient, cubic liquid-crystalline geometry, pore size and distribution, interface curvature, temperature, pH, and ionic strength of the release medium. The release mechanism of several hydrophilic model drugs from the cubic and reversed hexagonal liquid crystalline was investigated. These studies indicated that diffusion is the predominant mechanism of drug release, and the drug release rate from cubic ones is faster than the hexagonal liquid crystalline. Furthermore, the in vivo drug release profiles of ^14^C-glucose from cubosomes and hexagonal phase were consistent with the in vitro release profiles, which indicated the nanostructure of cubosomes and the nature of lipid could be utilised to control the release rate of hydrophilic drugs [28]. But it is difficult for the hydrophobic drug to escape from the cubosomes in vitro due to the affinity of the drug with the hydrophobic domain in the cubic phase. Hence, the release profiles of hydrophobic drug-loading cubosomes in distilled water media (pH 6.5) and digestion media (0.1 M Hydrochloric acid) were investigated and found that the drug release rate in the digestion media was drastically improved. Also, it is reported that the plasma concentration of Silymarin in vivo showed an increased drug release rate from cubosome formulation as compared to Legalon^®^, a commercial capsule formulation [29].

## 5. Advantages and Disadvantages of Cubosomes

The most essential advantages of cubosomes are their biocompatibility, the ability to be loaded with numerous medications, and their simplicity [18]. Due to unique qualities like thermostability, bioadhesion, the capacity to encapsulate medicinal compounds, and the capacity for regulated release, cubosomes are considered prospective carriers for diverse routes of administration [30]. Other advantageous characteristics of cubosomes include the solubilisation of lipophilic, hydrophilic or amphiphilic drugs, sustained release of incorporated drugs; bioadhesion; protection of drugs from degradation; and the non-toxic nature of the building blocks of the cubosomes. The advantages and disadvantages of cubosomes are shown in Table 2. A few examples of cubosome loaded with proteins and genetic materials are shown in Table 3.

## 6. Structure and Components of Cubosomes

**Figure 2:**
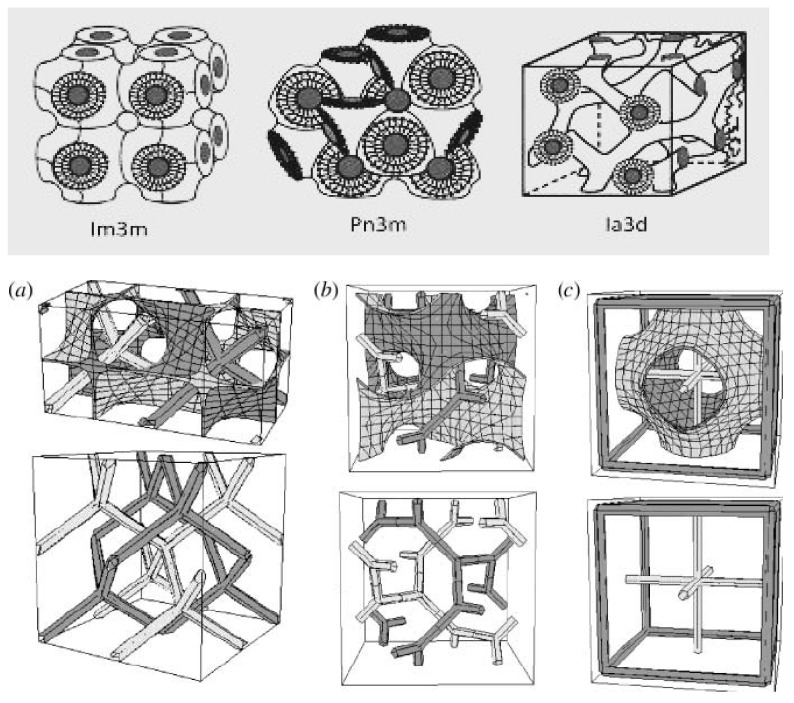
Underlying minimal surfaces and skeletal graphs (centre of water channels) for the inverse bicontinuous cubic phases. (**a**) Pn 3 m (Diamond), (**b**) Ia 3 d (Gyroid) and (**c**) Im 3 m (Primitive) [33].

Cubosomes are self-assembled liquid-crystalline particles that have solid-like rheology and are also bicontinuous cubic-phase liquid crystals. Because of their interesting bicontinuous topologies, the cubic phases have very high viscosity. The bicontinuous cubic liquid-crystalline phase is an optically transparent, viscous substance with a structure that is on the nanometer scale, as shown in Figure 2. Its geometric model for drug distribution was provided, constructed, and tested. The surfactant forms bilayers that are twisted into a three-dimensional, periodic, minimum surface, generating a tightly packed structure that looks like a honeycomb with water and lipid domains [18]. The presence of two types of cubic structures, D- and P-type [s9], was confirmed by small-angle X-ray scattering. D-type is dominant at a 3% concentration of P407, whereas the P-type diffraction pattern is barely discernible, implying that the polymer preferentially arises on the particle surface and the inside has a D-type structure dominated by GMO (Rylo MG 19 or Glycerol Monooleate).

On the micron scale, coarse cubosomes have the same Pn3m/QIID (Diamond or D-surface) morphology as their bulk cubic phase. In contrast, the Im3m/QIIP (Primitive, Schwarz, or P-surface) morphology dominates following homogenisation, which could be due to the additional polymer or other factors [32]. The structure helps to maintain the efficacy and stability of active ingredients like vitamins and proteins [32]. Cubosomes are thermodynamically stable and can persist for an infinite amount of time [33]. The addition of polymers to cubosome colloidal dispersions can help to stabilise them. They also have the potential for regulated delivery of actives, as the tortuous diffusion of the actives through the cubic phase’s “regular” channel structure governs diffusion [27]. The lipids that are used to prepare cubosomes are waxy and sticky, thus, incapable of forming small discreet particles. Noncohesive water-soluble starch coating on waxy lipids is discovered to prevent agglomeration and allows particle size control.

### 6.1. Amphiphilic Lipids

GMO or Rylo MG 19 or Glycerol Monooleate, also known as monoolein, and phytantriol (PHYT) are the most commonly employed amphiphilic lipids in cubosome synthesis at the moment [34,35]. Under excess water conditions, these commonly used lipids have a Pn3m/QIID (Diamond or D-surface) shape in temperatures ranging from room temperature to 43 °C and above 80 °C, respectively. These lipids are also biocompatible, have acceptable bulk phase characterisation, and were recently cleared for use in vivo [36].

#### 6.1.1. Glycerol Monooleate (GMO)

GMO is a polar, unsaturated monoglyceride with a melting point of 35–37 °C, having a hydrophilic-lipophilic balance (HLB) value of 3, and is clear and colourless in appearance. It is composed of oleic acid glycerides and other fatty acids, the most notable being monooleate. Monooleate is an amphiphilic lipid which may form lyotropic liquid crystals in a variety of shapes [37]. GMO has both hydrophilic and hydrophobic qualities owing to the existence of hydroxyl groups within the head region, which can form H-bonds with water in an aqueous solution and hydrocarbon chains in the tail [24]. Furthermore, GMO is a biodegradable, biocompatible and non-toxic substance classified as GRAS (generally recognized as safe) and included in FDA inactive ingredients guide, widely used as an emulsifier.

#### 6.1.2. Phytantriol (PHYT)

It is a common constituent in cosmetic products, used as an alternative to GMO in cubosome preparations. It has the ability to form a bicontinuous cubic structure in aqueous media under physiological conditions and temperature. Due to its high chemical stability, enhanced skin penetration properties, and improved moisture retention PHYT has gained more interest in the biomedical field. Also, it has the ability to sustain the release of various drug molecules, especially drugs having hydrophilic properties [38].

### 6.2. Stabilizers

When cubosomes are dispersed in aqueous media, the dispersed particles become kinetically unstable, as they tend to aggregate due to the exposure of hydrophobic portions to the external hydrophilic aqueous media. A surfactant is required to keep cubosomes colloidally stable and prevent them from re-coalescing into the bulk cubic phase. The stabiliser provides an electrostatic barrier between particles to prevent close contact of particles hence keeping the dispersed particles in a highly stable form. The most commonly used stabilising agents are Pluronics. Poloxamer 407 (F127), a PEO_99_–PPO_67_–PEO_99_ tri-block copolymer, is a widely utilised surfactant for the production of cubosomes, with the PPO parts arranged at the surface or within the bilayer structure, and the PEO chains exposed to the surrounding water phase. In the case of cubosomes, the stabilising technique of F127 appears to be different from that in the case of simple dispersions like emulsions. The stabilising effect of F127 is due to the result of the adsorption of a hydrophobic portion (PPO) onto the surface of the particles, while the hydrophilic portion (PEO) extends out into the aqueous media, providing steric shielding. The stabiliser interacts with the scattered particles’ structure and manipulates the phase behaviour in cubosomes [14]. Stabilisers are used with a concentration of up to 20% *w*/*w* depending on the dispersed particles. A few examples of lipids and stabilising agents used in the preparation of cubosomes are shown in Table 4.

## 7. Drug Loading in Cubosomes

An adequate amount of small-molecule drugs, peptides, biologics, or bioactives can be loaded onto the synthesized cubosomes. The three main mechanisms of loading the cargo include loading within the lipid bilayer, attaching to the lipid membrane, or localising the drug within the water channels in the cubic phase. The loading of the drug moieties could be achieved either by adding the therapeutic agent to the molten lipid or by co-lyophilising with the lipid film before dispersion. Alternatively, drug moieties could also be loaded after dispersion onto already prepared cubosomes by incubation method. Most of the small-molecule drugs, peptides, and proteins are loaded within the lipid bilayer. Additionally, cubosomes are synthesised by using single or binary lipid compositions, mainly comprising phytantriol and monoolein. Although drug loading can be quantified using a multitude of methods, small-angle X-ray scattering (SAXS) remains the most employed. Therefore, these studies revealed the potential of using cubosomes as a drug delivery modality, especially for the delivery of anticancer agents [39].

The major advantage of cubosomes over other particles, like liposomes, is their larger hydrophobic region, which allows a larger loading capacity of hydrophobic drugs while still permitting the loading of hydrophilic ones. The research revealed that curcumin in phytantriol cubosomes has a larger loading capacity compared to curcumin liposomes [40]. Furthermore, due to the cubosomes lattice structure, the particle membrane curvature can be tuned independently of its size. This characteristic is particularly important in the mimicry of highly curved structures, which are characterised by a higher membrane to surface area to volume ratio and increased membrane loading capacities [40].

The cubic structure of cubosomes has the ability to entrap the drugs and then release them based on different molecular weights and polarities, which follows the law of Higuchi-diffusion-controlled kinetics.

Q = [D_m_C_d_ (2A − C_d_)t]^½^

According to this equation, the release (diffusion) of agents from the matrix depends on the square root of time. Q is the quantity of the agents released per unit area of the matrix, D_m_ is the diffusion coefficient of the agents in the cubic matrix, C_d_ is the solubility of the agents in the matrix, A is the primary quantity of the drug per unit volume of the matrix, and t is the time. From this equation, the quantity and rate of drug release can be determined [41].

## 8. Methods of Preparation

Based on the energy sources employed to split the bulk phases, cubosome preparation methods can be divided into top-down and bottom-up approaches. On the one hand, top-down approaches use sonication and high-pressure homogenisation, whereas bottom-up strategies use hydrotropes to lower energy inputs. Poloxamer-407 (P407 or Pluronic F127 or PF127), a triblock polymer comprising of polyethylene oxide–polypropylene oxide–polyethylene oxide (PEO–PPO–PEO) copolymer, assists in stabilising lyotropic non-lamellar liquid-crystalline nanoparticles (LCNs) by preserving the liquid-crystalline inner structure of the nanoparticles and creating a steric barrier, is used in both approaches [42]. Comparison of various formulation techniques have been shown is Table 5.

### 8.1. Top-Down Approach

**Figure 3:**
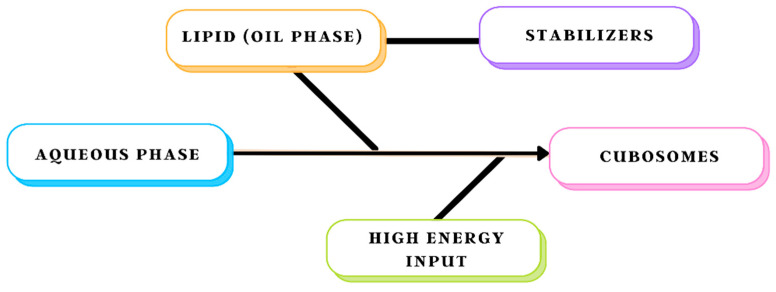
Schematic representation of the top-down approach method.

The most common approach for producing cubosomes is the top-down method, which involves two parts, as shown in Figure 3. The first step is to make a viscous bulk cubic phase by combining lipid with a stabiliser to prevent aggregation; the second step is to disperse the previous steps resulting in an aqueous medium using high-energy methods like high-pressure homogenisation or sonication, finally generating cubosomes. Although a bulk cubic phase resembles a translucent stiff gel composed of cross-linked water-swollen polymer chains, cubic phases are distinct because they are distinct thermodynamic phases with a periodic liquid-crystalline structure. For up to a year, this approach yields cubosomes that are impervious to aggregation. Vesicles, such as distributed nanoparticles of lamellar liquid-crystalline phase or vesicle-like structures, always coexist with cubosomes created via the top-down approach. At high oscillation frequencies, cubic phases become relatively elastic [43].

### 8.2. Bottom-Up Approach

**Figure 4:**
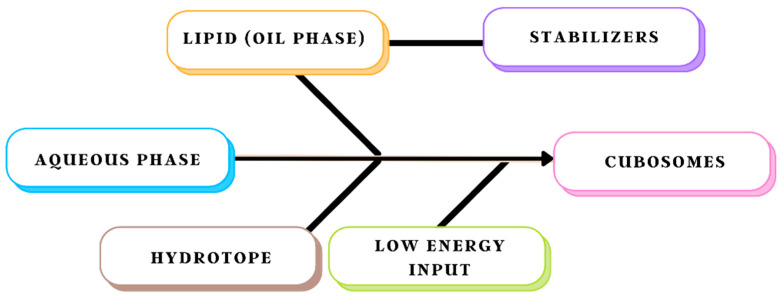
Schematic representation of the bottom-up approach method.

The bottom-up process starts with the creation of nanostructure basic building blocks, which are then put together to create the final product, shown in Figure 4. It is a more recently established method of cubosome production. Cubosomes can also be created at room temperature via a technique known as crystallisation from precursors. This procedure is referred to as the liquid precursor or solvent dilution method, according to Spicer et al. To make discrete nanoparticles, a polymer, a liquid crystal-forming lipid, and a hydrotrope are dispersed in surplus water with low energy input [27]. Hydrotrope is a vital component of the bottom-up strategy because it may prevent the formation of liquid crystals at high concentrations and break down water-insoluble lipids to make liquid precursors. Unlike the top-down technique, this dilution-based procedure can produce cubosomes without the requirement for time-consuming fragmentation. As a result, less energy is used. In addition, this approach is far more efficient in producing small particles. The method through which cubosomes originate could be the reason behind this. The top-down method is more like giant particle attrition, but the dilution-based method is more akin to small particles aggregating into larger particles, which is similar to using precipitation processes to generate nanoparticles [44].

The dilution-based strategy has certain distinct advantages over the top-down method when combining the different primary methods for creating cubosomes. For starters, it necessitates less energy input, avoiding lengthy fragmentation procedures. It can also be bonded with temperature-sensitive materials, which is an important differentiator. This technique is substantially more effective in creating smaller particles due to the unique production mechanisms of cubosomes. Finally, due to the homogenous dispersion of stabilisers onto the surface of nanostructured particles, the resulting cubosomes have long-term stability. Furthermore, using hydrotrope speeds up the set-up while producing cubosomes that are equivalent to or better than most of those created by other methods; also, the bottom-up method is best suited for scaling up to commercial batches [14]. Sherif et al. compared both approaches to discover that the bottom-up strategy results in small cubosomes with superior entrapment efficiency as well as slow dissolution rates. The bottom-up strategy, unlike the top-down approach, cannot successfully avoid the formation of vesicles [45].

**Figure 5:**
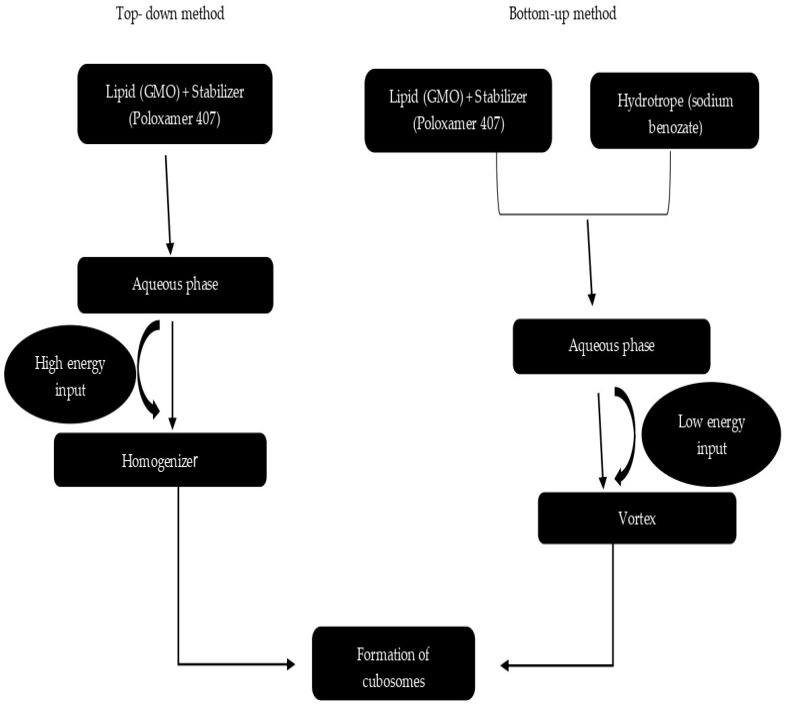
Diagrammatic representation of cubosomes preparation techniques.

Dispersion of cubosomes can be done by techniques like sonication, high-pressure homogenisation, spontaneous emulsification and spray drying. Sonication and high-pressure homogenisation provide the formation of complex dispersions containing vesicles and cubosomes with time-dependent ratios of each particle type. The difference between the top-down approach and the bottom-up approach is shown in Figure 5.

### 8.3. Heat Treatment

New hydrotrope-free cubosome manufacturing methods have recently been developed, which may help solve these problems. Muir et al. found that adding phosphate buffer saline (PBS) to a binary lipid system containing PYTH and a charged lipid, dodecyl dimethylammonium bromide (DDAB), can result in cubosome formation. When PBS is added to the DDAB, a charge-shield forms on the surface, changing the bilayer curvature and restoring the bicontinuous cubic phase. In this situation, heat treatment may be a viable choice. Heat treatment, in the strictest sense, is not an integrated method for the generation of cubosomes because it just facilitates the transition from non-cubic vesicles to well-ordered cubic particles [46]. As a result, a basic processing procedure that comprises a homogenisation and heat-treatment step can be used to create dispersed particles. Heat treatment minimises the small particle size fraction that corresponds to vesicles, resulting in the production of bigger cubic phases with narrow particle dispersion and strong colloidal stability, according to the research. When the full preparation procedure is considered, it is evident that the transition happens during the heat-treatment operation. Elevated temperatures could lead to a decrease in solubility and stability, which can be the cause of the transition. The surfactant has a high solubility when the temperature is below cloud point, allowing the particles to exist in a stable form with little fusion. When the surfactant solubility falls below a particular threshold, vesicles begin to fuse quickly [46].

### 8.4. Spray Drying

**Figure 6:**
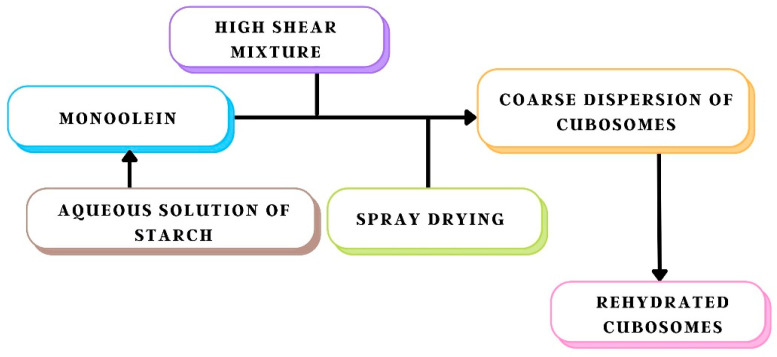
Schematic representation of the preparation of dry powder cubosomes.

Another method of cubosome preparation is the spray-drying process. Spray-dried encapsulated particles are made from an emulsion of liquid droplets or dispersions of solid particles in concentrated water-polymer solutions [47]. Both phases are sprayed through a curated nozzle, creating suspension droplets to collide with a dry, hot airflow. Excess water quickly evaporates, leaving dry powder particles made composed of the dispersed phase surrounded by an encasing of the previously dissolved polymer. The spray-drying process is easy to scale up and is now frequently utilised in consumer goods such as detergents and meals. Furthermore, the method makes it simple to preload actives into cubosomes before drying [40]. Finally, the polymer coating on the powder gives the hydrated cubosomes surface properties, which can be changed by identifying the perfect encapsulating polymer. The liquid feed can be changed to alter the resulting powder’s properties. For the production of starch-coated cubosomes powder precursors, high shear treatment of monoolein in aqueous starch solution produces a coarse cubosomes dispersion that is then pushed through a nozzle and dried. According to gravimetric measurements, drying removes approximately all of the water in the powder, resulting in a final composition of around 72% starch, 4% *w*/*w* water, and 24% monoolein in the finished powders [29], as shown in Figure 6.

**Table 5:** Comparison of formulation techniques.

Techniques	Benefits	Drawbacks	References
Bottom-up approach	Requires low energy input; hence it can be used safely for drugs that are temperature sensitive	This method can be preferred only for thermosensitive drugs, formulations are stable for a short period	[47]
Top-down approach	Reduces aggregation and improves the stability of formulations for up to one year	High-energy input is required to disperse the aggregates into cubosomes	[47]
Solvent evaporation method	Produce cubosomes of smaller particle size with higher physical stability	Due to the large-scale mixing of water and ethanol high polydispersity index is reported	[48]
Spray-drying method	A highly versatile, cheap and scalable method. The best method for drying labile products, such as proteins and vaccines	Difficulty in spray drying of the formulation as a cubic phase is formed immediately upon hydration of monoolein	[48]

## 9. Characterisation of Cubosomes

For compositions consisting predominantly of vesicle-forming bilayer lipids, DSC, NMR, and fluorescence microscopy have historically been used to detect phase transition borders and quantify fundamental material properties as a function of temperature and composition. Lipid phases are assigned via small-angle scattering (mostly X-ray). The lipid sample is bombarded with high-energy X-rays, and the ensuing diffraction pattern provides a discrete set of rings. This information is used to create the preponderance of phase diagrams as a function of lipid concentrations, hydration, pressure, and temperature. Using small-angle X-ray scattering, cryotransmission electron microscopy, or Cryo-TEM, is a strong tool for characterising soft matter dispersions and assigning phases (SAXS). Cryo-TEM was used to obtain high-resolution images of cubosomes, permitting direct imaging of the interior cubic phase structure for dispersion confirmation, surface structure information, and exact size estimation. The process of cryo-TEM entails passing a stream of electrons through an ultrathin object and reacting with it as they pass through. The interaction of electrons travelling through the specimen produces an image that is magnified onto a screen, such as a layer of photographic film, a fluorescent screen, or sensors to be detected. Cryo-TEMs can photograph at much higher resolutions since electrons have a smaller wavelength [49].

Sub-tomogram averaging, which was previously employed for protein structure reconstructions, has now been demonstrated to work on cubosomes. DLS, or dynamic light scattering, is a method for sizing and stabilising cubosome dispersions to ensure that they are monodisperse and that no aggregation occurs throughout the required periods. Although nanoparticle tracking analysis (NTA), which characterises single particles and generates a population of sizes rather than bulk measurements like DLS, is not widely in use for cubosomes, it has been used for vesicles and can be used to confirm the exact particle concentration as well as size information. For determining the dispersions’ particle size distribution, photon correlation spectroscopy may be performed. At a temperature of 250 °C, 100-s intervals are used to measure the refractive index (RI). Samples are diluted with water to change the signal level. The average particle size and the polydispersity index are computed [49,50].

### 9.1. Characterization of Non-Lamellar Liquid Crystalline

Various methodologies are utilised to characterise the physicochemical features of LCPs. These techniques were recently divided into two groups by Amar-Yuli et al.: direct techniques and indirect techniques [51]. Direct techniques include small-angle X-ray and neutron scattering, as well as optical and electron microscopy. Indirect approaches that provide extra information include spectroscopy, which includes nuclear magnetic resonance, dynamic light scattering, and rheology. A few strategies will now be discussed in greater detail.

#### 9.1.1. Electron Microscopy

Cryogenic transmission electron microscopy (cryo-TEM) allows for direct observation of samples in their hydrated state by vitrifying them in a thin film suspended between polymer-coated grids. Traditional (negative staining) transmission electron microscopy (TEM), in which materials are dried on carbon grids before being seen under the microscope, is not suggested because of the complications connected with dehydration. [49].

Cryo-TEM is a powerful supplement to scatter data since it allows for direct visualisation and verification of lattice symmetry. The gold standard for characterising the structure type of non-lamellar liquid-crystalline dispersions is a combination of cryo-TEM and scattering. Cubosomes are faceted cubic particles with a cubic shape. Cryogenic field-emission scanning electron microscopy (cryo-FESEM) has recently been reported as a useful supplementary imaging technique for studying the nanostructure of non-lamellar mesophases [51,52]. Dispersions can be seen in a frozen, close-to-natural form using cryo-FESEM. The reported data in studies by Rizwan et al. support differential geometry-based descriptions of lipid cubic phase nanostructures, especially cubosomes, in which a single continuous lipid bilayer is distorted to split space into two congruent and non-intersecting water channels. Cryo-FESEM revealed that the nanostructure of the dispersions was comparable to the microstructure of the non-dispersed phases [52].

Compared to transmission microscopy, one of the limitations of using scanning microscopy to probe the structure of submicron particles is the lower resolution (50–100 nm) and the potential formation of ice crystals during sample transfer [52,53]. Ice crystals are frequently large and can cover important areas. Frozen condensed water droplets, in addition to large ice crystals, can make data interpretation difficult and misleading. It has been demonstrated that plunge freezing in liquid propane reduces ice crystal size lower than the resolution of the microscope. Additionally, prior to coating, samples are usually sublimed for a few minutes to remove any unwanted surface ice [54].

#### 9.1.2. X-ray Scattering

Scattering techniques are essential for unambiguously establishing the structure of the mesophase of interest. Three types of radiation are commonly utilised in scattering studies: light, neutron and X-ray. By measuring the intensity of scattered X-rays at small angles, small-angle X-ray scattering (SAXs) investigations are frequently employed to explore structure at the mesoscale. Although SAXs is an invaluable tool for identifying mesophases, it is not without its demerits. Weak reflections when using a lab source are a problem with SAXs, especially in dispersed liquid-crystalline systems due to their small non-uniform crystallographic microstructure. Furthermore, some systems may have two or more coexisting mesophases, making it challenging to assign peaks to specific space groups [47]. SAXs is based on the Bragg formula [55]. Bragg’s law explains the relationship between an X-ray light shooting and its reflection from a crystal surface. It is useful for measuring wavelengths and determining the lattice spacing of crystals.

$$S=\frac{2sin\theta }{\lambda }=\frac{q}{2\pi }$$

where *S* is the scattering vector, *q* is the scattering factor, θ is the scattering angle, λ is the wavelength of the X-ray [55].

### 9.2. Particle Size Distribution

Particle size is determined by dynamic laser light scattering (DLS) using a zeta sizer (photon correlation spectroscopy). It is a simple, non-invasive method to characterise the particles in suspension. The sample diluted with a suitable solvent is adjusted to a light scattering intensity of about 300 Hz and measured at 25 °C in triplicate. The data is collected and shown by using average volume weight size [56]. The major drawback of DLS measurements is that larger and heavier particles contribute strongly to the overall mean decay rate of a poly-disperse solution, often leading to an overestimation of such larger particles. Another important parameter to be determined for liquid-crystalline systems is the zeta potential. Zeta-potential measurements can only be performed indirectly, where the velocity of a charged particle that moves under the influence of an applied electric field (electrophoretic mobility) can be calculated. For pharmaceutical applications, to determine the existence of cationic or anionic particles in solution, it is crucial to measure the zeta potential. [57]. Polarising microscopy can be used to determine the morphology of liquid crystalline based on the optical birefringence phenomena of liquid crystalline [58].

### 9.3. Entrapment Efficiency

Entrapment efficiency and drug loading of cubosomes can be accessed using chromatography techniques, dialysis, small-angle X-ray scattering, or ultra-filtration techniques. The amount of unentrapped drug can be further analysed using a UV spectrophotometer, HPLC analysis, and fluorescence correlation spectroscopy. The unentrapped drug concentration is determined, which is subtracted from the total drug added in the formulation, and the amount of drug is analysed by using a spectrophotometer or radioactivity [59].

### 9.4. Measurement of Drug Release

The drug release mechanism from cubosomes is based on the principle of drug diffusion, and the drug concentration gradient across the cubosomes is the driving force for diffusion. The factors influencing the drug release rate are the solubility of the drug, diffusion and partition coefficient, cubic liquid-crystalline geometry, pore size and distribution, temperature, pH and ionic strength of the release medium. The drug release from cubosomes can be evaluated by the pressure ultra-filtration method [60].

### 9.5. Stability Studies

The physical stability can be studied by investigating the organoleptic and morphological aspects of cubosomes as a function of time. Particle size distribution, zeta potential, drug content and entrapment efficiency of cubosomes at various temperatures can be determined at different time intervals to evaluate the possible variations by time [61]. The phase transition in liquid crystalline is accompanied by exothermic or endothermic energy changes. To investigate the stability of liquid crystalline, DSC can be used to determine the phase transition temperature of the binary liquid-crystalline system. Also, the viscosity of cubosome formulations must be determined at different angular velocities using a rotary viscometer [62].

## 10. Applications of Cubosomes

Cubosomes, which are bicontinuous cubic phase liquid crystals, have a number of characteristics that make them a possible universal medium for the transport of diverse therapeutic actives. Like conventional drug delivery systems, these nanoparticles utilise surfactant and/or polymer systems to create supra-assemblies, frequently used as active transport vesicles. Adequate concentrations of small-molecule drugs must be loaded for the cubosomes to act as viable drug delivery vehicles. Cubosomes are used to deliver anticancer medications in several investigations, with encapsulation efficiencies ranging from 71 to 103%. As a result, these investigations highlight the potential of cubosomes as a drug delivery vehicle, particularly for anticancer drugs [63]. Pharma companies are trying to use cubosomes as stabilisers and pollutant adsorbents in cosmetics. They have been formulated as cosmetic products like skincare, hair care, and antiperspirants, and a few companies have filed patents too. Alpha-lipoic acid (ALA) is a naturally occurring fatty acid with a potent antioxidant activity which exists in the mitochondria of all kinds of prokaryotic and eukaryotic cells. A recent study has demonstrated that the formulation of ALA in cubosome dispersion showed excellent results in reducing facial lines with almost complete resolution of fine lines in the periorbital region and upper lip area with improved skin texture and colour in volunteers.

To overcome the obstacles in dispensing these bio-macromolecular medications, such as interference by membrane barriers and drug instability, a variety of drug delivery protocols have been developed in recent years. The development of highly effective active drug carrier systems is accelerated by the synergistic integration of different nanoparticles with target ligands. Recent research has found parallels between the bicontinuous structures created in human skin layers and those found in cubic phases, promising a better understanding and treatment of skin transport. Cubosome-based triglyceride–monoolein combinations paired with the antibiotic metronidazole have been developed as commercial applications for periodontal disease. The lipid–drug mixture is applied to the gums of the mouth. When it comes in contact with the saliva, it hydrates to form a bulk cubic phase, which subsequently distributes the drug in a uniform manner. Cubosomes have the ability to target diseases at the location of the problem and can circulate in the body after being injected. Cubosomes are particularly useful in cancer therapy because of this property. When it comes to cancer targeting, the size of the delivery system is crucial because of the increased permeability and retention effect. Examples of cubosomes for various drug delivery are shown in Table 6, Table 7, Table 8, Table 9 and Table 10. Attaching ligands (proteins, folic acid derivatives, peptides) to cubosome surfaces can also be used to target diseased cells [63,64].

Cubosomes have been considered an excellent candidate for oral drug delivery. They enhance the oral bioavailability of poorly water-soluble drugs. cubosome formulations containing the protein ovalbumin were developed with a high entrapment efficiency and slow-release behaviour in vitro, showing the potential of cubosomes as a novel vaccine delivery system. Additionally, the unique crystalline structure of cubosomes protects the entrapped drug from degradation in the gastrointestinal tract [65].

The drug contained in cubosomes can easily penetrate the epidermis of mucosal and skin, resulting in an improved bioavailability of drugs. Cubosomes are used as carriers for dexamethasone and flurbiprofen for ocular treatment, and the studies revealed that the apparent permeability and the bioavailability of these drugs were greatly increased [66].

Cubosomes have been developed as an excellent delivery system due to their unique solubilisation, high encapsulation, sustained release behaviour and in vivo stabilisation. It is demonstrated that the terminal half-life of somatostatin cubosomes given as an intravenous injection in rats was significantly improved as compared to the corresponding somatostatin solution [67].

Due to their unique structure, cubosomes provide a promising vehicle for transdermal drug delivery. Cubosomes and microneedles have been used as a synergistic approach for vaccine delivery through the skin. Results showed that the use of microneedles enhanced the permeation of the aqueous peptide mixture through the skin layers, and cubosomes showed longer retention of the formulated peptide within the skin. The topical delivery of cubosomes is based on the unique properties of liquid crystals. Due to their bioadhesive nature, the liquid crystal systems facilitate the delivery of drugs to mucosal surfaces like the buccal, ophthalmic and vagina [68].

**Table 6:** Marketed lipid-based products in clinical development.

Products	Drugs	Target Diseases	Status	References
SPI-077 (Alza)	Cisplatin	Solid tumours	Phase II (Development terminated)	[69]
CPX-351 (Celator)	Cytarabine:daunorubicin	Acute myeloid leukaemia	Phase II	[69]
CPX-1 (Celator)	Irinotecan HCI:floxuridine	Colorectal cancer	Phase II	[70]
Brakiva (Talon)	Topotecan	Relapsed solid tumours	Phase I	[71]
Lipoplatin (Reglon)	Cisplatin	Non-small cell lung cancer	Phase III	[71]
ThermoDo x(Cesion)	Thermosensitive doxorubicin	Primary hepatocellular carcinoma	Phase III	[72]
Exparel (Pacira)	Bupivacaine	Nerve block	Phase II	[73]
Stimuvax (Oncothyreon/Merck)	Anti-MUC1 cancer vaccine	Non-small cell lung cancer	Phase III	[73]

**Table 7:** List of drugs loaded in cubosomes for anticancer drug delivery.

Active Ingredients	Polymers Used	Applications	References
20 (S)-protopanaxadiol, Piperine	Monoolein (MO), Poloxamer 407 (PF127)	Drug Delivery	[74]
3-bromopyruvate	Monoolein (MO), Poloxamer 407 (PF127), Folic acid	Tumour Targeted Delivery	[75]
Camptothecin	Squarain-based NIR-emitting fluorescent probe, Pluronic F108 (PF108), Monoolein	Theranostic and Bioimaging	[75]
Curcumin	Polyethylene glycol 400 (PEG-400), RH40, and Monoolein (MO)	Anticancer activity	[76]
Doxorubicin (DOX)	Monolinolein, Pyridinylmethyl linoleate	Tumour Targeted Delivery	[76]
Gambogenic acid	Monoolein (MO)	Drug delivery in cancer therapy	[77]
Meso-Tetraphenylporphine-Mn (III) chloride	Monoolein (MO), Polyethylene glycol (PEG), Phospholipids	Bioimaging	[77]
Metformin	Monoolein (MO), Poloxamer (Pol.) 407 (PF127)	Drug Delivery	[78]
Paclitaxel (PTX)	Monoolein (MO), Poloxamer 407 (PF127), Polyethylene glycol (PEG)	Drug Delivery	[78]
Pemetrexed and Resveratrol	Monoolein (MO)	Drug Delivery in lung cancer	[79]
Dacarbazine	GMO, Pol. 407	First-line chemotherapy medication against melanoma	[79]
5-fluorouracil (5-FU)	GMO, Pol. 407	For the treatment of advanced gastrointestinal cancers, including hepatocellular carcinoma	[80]
20 (S) protopanaxadiol (PPD)	GMO, Pol. 407	Anticancer drug	[80]
Folic-acid-modified etoposide cubosomes	Polyethylene glycol 400 (PEG-400), RH40, and Monoolein	Breast cancer	[81]
Cisplatin- and paclitaxel-loaded cubosomes	Monoolein (MO), Poloxamer 407 (PF127), Polyethylene glycol (PEG)	Liver cancer	[81]
Icariin cubosomes	Monoolein (MO), Poloxamer (Pol.) 407 (PF127)	Ovarian Cancer	[81]
Cisplatin and metformin nanocubosomes	Monoolein (MO), Poloxamer (Pol.) 407 (PF127)	Colorectal cancer	[82]

**Table 8:** Applications of cubosomes as ocular drug delivery system.

Loaded Drug	Lipids & Stabilisers	Therapeutic Uses	References
Dexamethasone (DEX)	GMO, Pol. 407	Treatment of anterior ocular inflammation	[83]
Flurbiprofen (FB)	GMO, Pol. 407	For treatment of ocular inflammation	[83]
Ketorolac	GMO, Pol. 407	For treatment of ocular symptoms due to allergies	[84]
Timolol (TM)	GMO, Pol. 407	Non-selective beta-blocker drug for the treatment of glaucoma	[84]
Cyclosporine A	GMO, Pol. 407	Immunosuppressive agent for treating inflammatory and immune-related ocular diseases	[85]
Pilocarpine	GMO, Pol. 407	To treat open-angle glaucoma and acute angle-closure glaucoma	[85]

**Table 9:** Dermatological applications of cubosomes.

Loaded Drug	Oil Stabiliser	Therapeutic Use	References
Capsaicin	GMO, PYT, Pol. 407	Used in the treatment of psoriasis, pruritus, and contact allergy	[86]
Silver sulfadiazine	GMO, Pol. 407	Used for the treatment of infected burns	[86]
Indomethacin	GMO, Pol. 407	Anti-inflammatory drug	[87]
Hydroxypropyl β cyclodextrin/minoxidil complex	GMO, Pol. 407	Minoxidil for hair growth	[87]
Antimicrobial peptide (AMP) LL-37	GMO, Pol. 407	Used for treatment of skin infection caused by Staphylococcus aureus	[88]
Erythromycin	GMO, Pol. 407	Treatment and prevention of several types of acne as a result of its bacteriostatic activity against Propionibacterium acnes	[89]
Dapsone	GMO, Pol. 407	For treatment of acne, leprosy & systemic lupus erythematosus	[89]
Vaccination through transcutaneous immunization (TCI)	Microneedle enhances the permeation of the peptide mixture in water through the skin layers, and cubosomes with peptide showed longer retention within the skin	Microneedles (MNs) and cubosomes have been used successfully as a synergistic method for the delivery of vaccines via skin	[90]

**Table 10:** Oral drug delivery utilising cubosomes formulation.

Loaded Drug	Lipids & Stabilisers	Therapeutic Uses	References
Insulin	GMO, Pol. 407	For treating type 1 diabetic-induced rats (insulin-dependent diabetes)	[91]
Ibuprofen	PYT, Pol. 407	Non-steroidal anti-inflammatory drug with analgesic properties	[92]
Simvastatin	GMO, Pol. 407	For cholesterol control in the body	[92]
Piperine	GMO, Pol. 407 with Tween 80 and Cremophor RH 40	Natural alkaloids with memory-enhancing potentials used in the treatment of Alzheimer’s disease (AD)	[92]
Amphotericin B	PYT, Pol. 407	For several types of fungal infections, such as histoplasmosis and Leishmaniasis	[93]

Table 11 describes the list of drugs that are incorporated into cubosomes for their effective and targeted delivery against various conditions.

## 11. Conclusions

Cubosomes are being widely explored and are attracting a lot of attention, especially in preclinical investigations, because of their enticing features that are regarded as perfect for successful drug administration. However, numerous obstacles and constraints must be overcome before these cubosomes can be successfully implemented in a therapeutic context. Cubosomes have a substantially greater bilayer area to volume of particles than liposomes or vesicles with a more substantial viscous barrier to rupture. Recent breakthroughs have made it possible to rationally develop smart cubosome systems for a variety of applications. Cubosomes offer a lot of potential as a medication delivery system for a variety of medicinal treatments [101].

Solubilisation of poor water-soluble medicines, along with the regulated and prolonged release of loaded actives, are two primary advantages of their use as delivery carriers. Cubosomes can be administered in a variety of ways, including intravenous, intranasal, oral, ophthalmic, and topical routes, due to their excellent qualities. One of the most distinguishing characteristics of cubosomes is their bioadhesive nature, which allows them to be used in topical as well as mucosal formulations for the administration of various drugs. Material scientists are interested in the cubic phases’ twisted but regular structure as a blueprint for complicated solid materials. An intimate understanding of stabiliser–membrane interactions, demonstrations of pore size analogous to bulk phase work, cytotoxicity studies with the study of interaction mechanisms, along with the demonstration of smart release is all extraordinary characteristics that shall, without a doubt, enhance the applications of cubosomes. They are transitioning into next-generation lipid nanoparticles as the fundamental knowledge base grows. Furthermore, cubosomal nanoparticles are being considered potential nano vehicles for loading and delivering peptides and protein-based medicines, but the reported research studies are still in the early stages. Cubosome has established itself as a viable technology platform with widespread clinical acceptability.

The oral application of cubosomes showed that they can be used effectively to increase the absorption of poorly water-soluble drugs and protects the liable drug from enzymatic degradation and in targeted drug delivery. They provide a promising vehicle for effective transdermal drug delivery with enhanced skin permeation and low irritation potential. Cubosomes are used for the delivery of anticancer drugs with reduced serious side effects of the chemotherapeutic agents and targeted drug delivery. They have been approved as an effective ocular drug delivery system with enhanced ocular residence time and bioavailability without irritating the eye [102].

Apart from all of these benefits of cubosomes, there are still some major outstanding challenges that need to be addressed. It includes further enhancing the applications of cubosomes and a deeper understanding of stabilisers as well as membrane interactions. Several other parameters related to the structural characteristics of the cubosomes, like the encapsulation efficiency, drug release profiles, dosing frequency, and compatibility of the cubosomal components with the blood fluids, as well as with commonly administered medications in co-morbid patients, should be scrupulously examined both in vitro and in vivo. In addition, large-scale production of cubosomes is sometimes difficult due to high viscosity, and there is a low entrapping of water-soluble components due to the presence of a high volume of water inside the cubosomes. Although great progress has been made, more extensive in vitro and in vivo cytotoxicity studies, including the mechanism of interaction with cancer cells, need to be further addressed in utilising the cubosomal formulations in the clinical setting.

## Figures and Tables

**Table 2:** Advantages and disadvantages of cubosomes [31].

Advantages	Disadvantages
Cubosomes are biocompatible, biodegradable, non-irritating and thermodynamically stable	Water-soluble drugs are less likely to be entrapped since they contain a large amount of water
Lipophilic, hydrophilic, and amphiphilic medicines can all be loaded into cubosomes	Large-scale production of cubosomes is challenging due to their high viscosity
They have a high drug-loading capacity due to their large interior surface area	Potential to leak during the storage or in vivo transmission
With 3-D nanostructures with both hydrophilic and hydrophobic domains, cubic liquid-crystalline phases are being employed as drug delivery systems in medical therapeutics	Phase change is possible if cubosomes are exposed to the external environment
While lipid ingredients are biocompatible, bioadhesive, and digestible, the huge interfacial surface can provide a complex diffusion pathway for the continuous release of entrapped drug molecules	Possibility of particle growth if the particles are left alone for a long time
The preparation process is simple. They are good solubilisers when compared to other lipid-based carriers. Increase the bioavailability of water-soluble peptides	

**Table 3:** Examples of cubosomes loaded with genetic materials and proteins [31].

Loaded Active Compound	Formulation
siRNA	Monoolein, DOTAP
siRNA	MO, DOTAP, MO-PEG
siRNA	Phy, DOTAP, F127 stabiliser
siRNA	DOTAP or DDAB, F127 stabiliser
Salmon sperm DNA	MO/PEG-15 Cocopolyamine
Thermomyces lanuginosus lipase	Monoolein, F127 stabiliser
Beta casein	MO/Phy, F127 stabiliser
Nerve growth factor	MO, beta casein stabiliser
Dopamine D2Lreceptor (membrane protein)	Ni(II) chelated EDTA amphiphiles
Cholera toxin B subunit	Phy, GM1, F127 stabiliser
Ovalbumin	MO/Phy, F127 stabiliser
Outer membrane protein F (OmpF)	Monolinolein, octyl-POE stabiliser
Human recombinant brain-derived neurotrophic factor (BDNF)	MO, eicosapentaenoic acid, PEG stabiliser

**Table 4:** Lipids and stabilizing agents used in cubosome preparation [38].

Stabiliser	Lipids
Monoolein	Laponite, Pluronic F 127, Modified starch
Monoelaidin	Pluronic F127
Monoolein or phytantriol	Pluronic F108
Sodium octyl sulphate (SCS)	Arginine-based cationic surfactant
Phytantriol	Myrj 59
β-XP (1-*O*-phytanyl-β-d-xyloside)	Pluronic F127

**Table 11:** Examples of drugs embedded in cubosomes and the outcome of the study.

Drugs	Objective of Study	Outcome of Study	References
Antimicrobial peptide LL-37	The antimicrobial potential of cubosomal LL-37 was evaluated using in vitro and ex-vivo skin irritation models.	The formulation provides superior protection to LL-37 against enzymatic degradation and significant bactericidal effects.	[94]
Erythromycin	Topical delivery of erythromycin for the treatment and prevention of acne.	The prepared cubosomes were effective in the topical delivery of erythromycin in a non-invasive and sustained manner.	[94]
Ketorolac	Monoolein and poloxamer cubic nanoparticles for ocular delivery of ketorolac.	Formulated cubosomes loaded with Ketorolac provided trans-corneal permeation and retention.	[95]
Simvastatin	Enhanced bioavailability of simvastatin to lower bad cholesterol and fats.	Prepared cubosomes enhanced the bioavailability of the lipophilic simvastatin when administered orally.	[95]
Indomethacin	Evaluation of Indomethacin-fabricated cubosomes for anti-inflammatory activity.	Homogenised monoolein and poloxamer-containing cubosomes prolonged the delivery of the drug.	[96]
Piperine	Evaluation of the memory-enhancing potentials (used in Alzheimer’s disease).	Prepared cubosomes were found to be safe with superior effects over free drugs and were effectively restoring cognitive functions.	[96]
Flurbiprofen (FB)	A non-steroidal anti-inflammatory drug used for the treatment of ocular inflammation.	The formulation showed less ocular irritation and enhanced transcorneal permeation of FB.	[18]
Insulin	Tested against the C-Type-1-diabetic-induced rat (insulin-dependent diabetes).	Cubosomes protected the loaded insulin against proteolysis. It was found to be stable at normal temperatures and controlled hyperglycaemia in a reproducible manner.	[18]
Dacarbazine	To reduce the side effects of melanoma.	Dacarbazine delivered through cubosomes decreases the side effects of intravenous delivery. It also enhanced the drug efficacy, safety, and shelf life.	[97]
20(S) protopanaxadiol	To evaluate the anticancer activity.	Cubosomes improved the oral bioavailability of the drug as a result of enhanced absorption.	[98]
Timolol	Synthesis of timolol-loaded cubosomes and their evaluation.	The prepared cubosomes showed increased corneal penetration, prolonged precorneal retention time and enhanced intraocular pressure lowering effect than the commercially available eye drops.	[99]
Docetaxel	Synthesis and evaluation of controlled release cubosomes incorporated with docetaxel as a thermosensitive depot.	The depot showed gradual drug release, preparation was free flowing at room temperature.	[100]

## Data Availability

Not applicable.

## Abbreviations

DOTAP	1,2-dioleoyl-3-trimethylammonium-propane
MO-PEG	Monoolein—poly(ethylene glycol)
DDAB	Dimethyldioctadecylammonium bromide
GM1	Beta-galactosidase-1
POE	Polyolefin elastomers
PPO	Polyphenylene oxide
DSC	Differential scanning colorimetry
NMR	Nuclear magnetic resonance
TEM	Transmission electron microscopy
Pol. 407	Polaxamer 407
PEG	Polyethylene glycol
